# Epidemiology of *Mycoplasma pneumoniae* Infections in Japan and Therapeutic Strategies for Macrolide-Resistant *M. pneumoniae*

**DOI:** 10.3389/fmicb.2016.00693

**Published:** 2016-05-23

**Authors:** Tsutomu Yamazaki, Tsuyoshi Kenri

**Affiliations:** ^1^Wakaba Children’s ClinicSaitama, Japan; ^2^Laboratory of Mycoplasmas and Haemophilus, Department of Bacteriology II, National Institute of Infectious DiseasesTokyo, Japan

**Keywords:** *Mycoplasma pneumoniae*, community-acquired pneumonia, infectious diseases surveillance, periodic epidemics, P1 cytadhesin, P1 typing, hemadsorption, macrolide resistance

## Abstract

Pneumonia caused by *Mycoplasma pneumoniae* (*M. pneumoniae* pneumonia) is a major cause of community-acquired pneumonia worldwide. The surveillance of *M. pneumoniae* pneumonia is important for etiological and epidemiological studies of acute respiratory infections. In Japan, nation-wide surveillance of *M. pneumoniae* pneumonia has been conducted as a part of the National Epidemiological Surveillance of Infectious Diseases (NESID) program. This surveillance started in 1981, and significant increases in the numbers of *M. pneumoniae* pneumonia patients were noted in 1984, 1988, 2006, 2010, 2011, 2012, and 2015. The epidemics in 2011 and 2012 were particularly widespread and motivated researchers to conduct detailed epidemiological studies, including genotyping and drug resistance analyses of *M. pneumoniae* isolates. The genotyping studies based on the *p1* gene sequence suggested that the *p1* gene type 1 lineage has been dominant in Japan since 2003, including the epidemic period during 2011–2012. However, more detailed *p1* typing analysis is required to determine whether the type 2 lineages become more relevant after the dominance of the type 1 lineage. There has been extensive research interest in implications of the *p1* gene types on the epidemiology of *M. pneumoniae* infections. Serological characterizations of sera from patients have provided a glimpse into these associations, showing the presence of type specific antibody in the patient sera. Another important epidemiological issue of *M. pneumoniae* pneumonia is the emergence of macrolide-resistant *M. pneumoniae* (MRMP). MRMPs were noted among clinical isolates in Japan after 2000. At present, the isolation rate of MRMPs from pediatric patients is estimated at 50–90% in Japan, depending on the specific location. In view of the situation, Japanese societies have issued guiding principles for treating *M. pneumoniae* pneumonia. In these guiding principles, macrolides are still recommended as the first-line drug, however, if the fever does not subside in 48–72 h from first-line drug administration, a change of antibiotics to second-line drugs is recommended.

## Surveillance of *M. pneumoniae* Pneumonia in Japan

**FIGURE 1:**
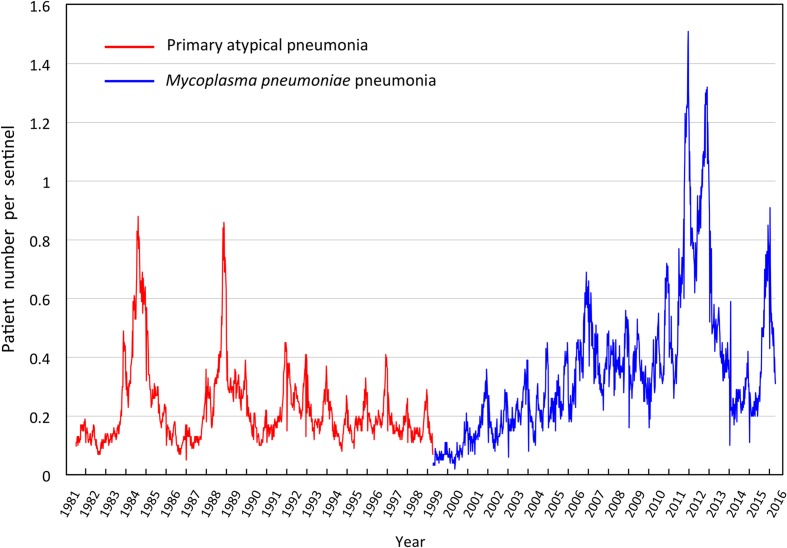
**Weekly cases of primary atypical pneumonia (from April 1981 to March 1999, red line) and *Mycoplasma pneumoniae* pneumonia (from April 1999 to present, blue line) in Japan reported by The National Epidemiological Surveillance of Infectious Diseases (NESID)**.

**FIGURE 2:**
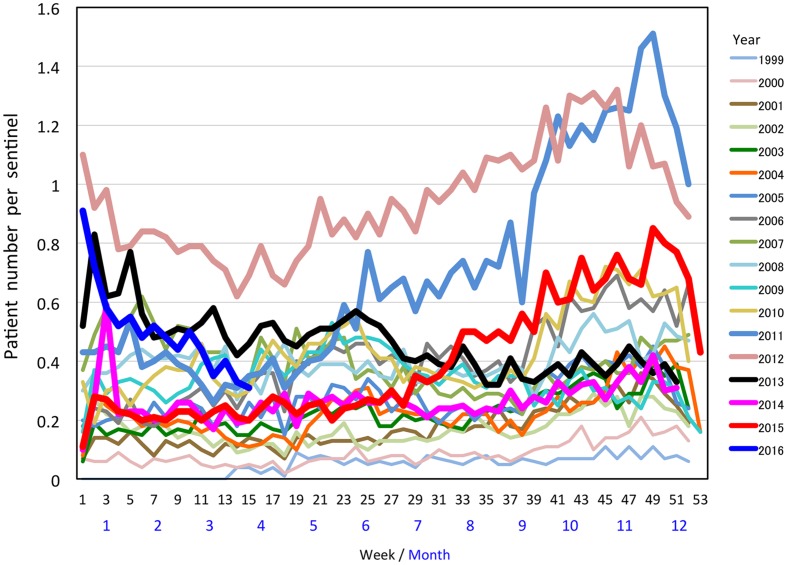
**Weekly cases of *M. pneumoniae* pneumonia in Japan between 1999 and 2015 reported by the NESID.** http://www.nih.go.jp/niid/ja/10/2096-weeklygraph/1659-18myco.html.

In Japan, the National Epidemiological Surveillance of Infectious Diseases (NESID) program is conducted under the Infectious Diseases Control Law (Law Concerning the Prevention of Infectious Diseases and Medical Care for Patients of Infections), which includes nationwide surveillance of pneumonia cases caused by *Mycoplasma pneumoniae*. *M. pneumoniae* pneumonia is classified as a category V infectious disease in the NESID, and the numbers of affected patients (total of outpatients and inpatients) are reported weekly from sentinel hospitals. Approximately 500 hospitals across Japan that have departments of pediatrics and internal medicine and more than 300 beds are currently selected as the sentinels for surveillance of *M. pneumoniae* pneumonia in Japan. For notification of each new *M. pneumoniae* pneumonia patient, confirmation is required using one of the tests listed in **Table 1** in addition to clinical symptoms observed by a clinician. Previously, culture isolation of *M. pneumoniae* and detection of serum antibodies against *M. pneumoniae* were employed as the tests for notification. However, detection of *M. pneumoniae* genomic DNA by polymerase chain reaction (PCR) or loop-mediated isothermal amplification (LAMP) and detection of *M. pneumoniae* antigens by immuno-chromatographic methods have been recently included in the tests for notification^1^. The data from sentinels are integrated at the Infectious Disease Surveillance Center (IDSC), National Institute of Infectious Diseases (NIID) and published weekly^2^. Since the NESID program was initiated in July 1981, the surveillance of primary atypical pneumonia (PAP) was continuously performed until March 1999. The criteria of PAP include pneumonia other than *M. pneumoniae* pneumonia, such as that caused by *Chlamydophila pneumoniae*, *Legionella pneumophila*, or several viruses. However, as the major cause of PAP is *M. pneumoniae*, this surveillance largely represented the general trend of mycoplasma epidemics. As of April 1999, *M. pneumoniae* pneumonia-specific surveillance was initiated by NESID under the revised Infectious Diseases Control Law. **Figure 1** shows the most recent *M. pneumoniae* pneumonia surveillance data collected by the NESID. In the early period of data collection, there were large increases of PAP patients observed in 1984 and 1988. Before the NESID surveillance was started in Japan, an extensive epidemiological study of *M. pneumoniae* pneumonia in school children was performed in the 1960s and 1970s in Sendai city (Niitu, 1984). In this study, an increase of *M. pneumoniae* pneumonia patients was observed every 4 years (i.e., 1964, 1968, 1972, and 1976), suggesting periodicity in the epidemics of this disease. Epidemics observed by the NESID in 1984 and 1988 (**Figure 1**) are compatible with this 4-year periodicity pattern observed in Sendai city. Given that these 4-year-cycle epidemics occurred in Olympic years, *M. pneumoniae* pneumonia has often been referred to as “Olympic disease” in Japan. However, after this period, 4-year-cycle epidemics were no longer observed in the NESID surveillance, although slight increases in the number of patients were observed in 1992 and 1996. The reason for disappearance of periodic epidemic is unknown, however, it is noteworthy that clarithromycin has been introduced for treatment of PAP since 1991. After 2000, *M. pneumoniae* pneumonia epidemics were observed in 2006, 2010, 2011, and 2012. The epidemics in 2011 and 2012 were particularly widespread and attracted public attention. Although the reason for these large epidemics in 2011 and 2012 is unknown, large increases in the numbers of *M. pneumoniae* pneumonia patients were also reported in Europe and other countries during this period (Chalker et al., 2011; Blystad et al., 2012; Nir-Paz et al., 2012; Pereyre et al., 2013; Kim et al., 2015). After these large epidemics in 2011 and 2012, the number of *M. pneumoniae* pneumonia patients decreased rapidly, and was quite low in 2014. However, the number of patients increased again since the summer of 2015 and reached a higher level during the winter (**Figures 1** and **2**). An increase of patient number was also reported in China in 2015 (Yan et al., 2016).

**Table 1:** Tests required for notification of *Mycoplasma pneumoniae* pneumonia from sentinel clinics.

Test (Method)	Specimen
Isolation of *M. pneumoniae* (Culture method)	Specimens derived from the patient’s respiratory tract
Detection of *M. pneumoniae* antigen (Immuno-chromatographic method)^a^	
Detection of *M. pneumoniae* DNA (PCR, LAMP, etc.)^b^	
Detection of antibody (serological diagnosis)	Serum

*^a^Four commercial immuno-chromatographic diagnosis kits for M. pneumoniae have been approved and used in Japan since 2013: Prime check Mycoplasma^TM^ (Alfresa Parma), Ribotest Mycoplasma^TM^ (Asahi Kasei), Prorast Myco^TM^ (LSI Medience), and Immuno Ace Mycoplasma^TM^ (Tauns Laboratories). The detection targets of these four kits are P1 adhesin, ribosomal L7/L12, DnaK and P30 proteins, respectively. ^b^Genomic DNA detection tests have been introduced to the surveillance since 2011.*

**FIGURE 3:**
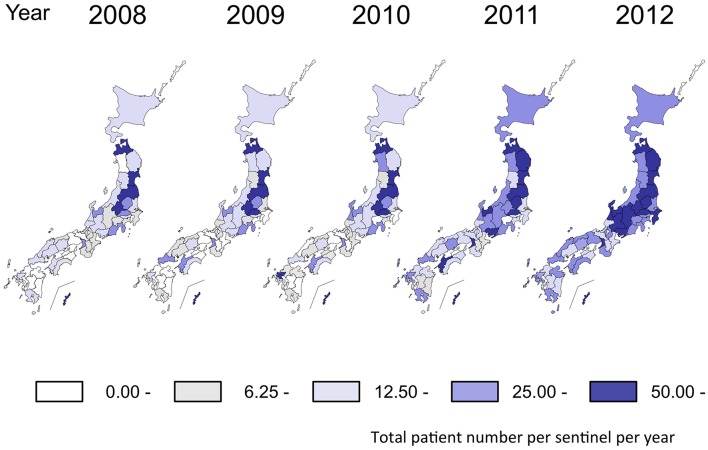
**Incidence of *M. pneumoniae* pneumonia in Japan by prefecture reported by the NESID.** The ranges of the number of cases in each prefecture (annual cases per sentinel) are shown in different colors. The figure is adapted from Infectious Agents Surveillance Report (IASR), 33 (10), 2012. http://www.nih.go.jp/niid/en/iasr-vol33-e/865-iasr/2738-tpc392.html.

Such period occurrences of *M. pneumoniae* pneumonia epidemics in 3–7-year intervals has been observed in surveillances of *M. pneumoniae* pneumonia in many areas of the world (Lind and Bentzon, 1976; Foy et al., 1979; Lind et al., 1997; Rastawicki et al., 1998; Ito et al., 2001; Eun et al., 2008; Chalker et al., 2011; Blystad et al., 2012; Youn and Lee, 2012; Kim et al., 2015). This phenomenon is considered to be one of the characteristic features of this disease. One potential reason for the pattern of periodic epidemics of *M. pneumoniae* pneumonia may be related to interactions between the pathogen and the immunological status of the human population (Fernald and Clyde, 1970; Mogabgab et al., 1975; Hayatsu et al., 1981; Leith et al., 1983; Barile et al., 1994). Once *M. pneumoniae* pneumonia epidemics occur, protective immunity may arise in the human population. However, this protective immunity may not last long, and the next generation will be dominated by younger individuals who had not been exposed, and thus do not have the protective immunity. In this situation, *M. pneumoniae* become active and may cause the next epidemic. A mathematical model of this process was recently reported (Omori et al., 2015). There are also reports of weather factors that might affect *M. pneumoniae* infections (Onozuka et al., 2009; Onozuka and Chaves, 2014). Examination of the NESID data from a seasonal perspective shows that the number of *M. pneumoniae* pneumonia patients generally increases in autumn and winter every year. However, depending on the year, small increases are sometimes also observed in early summer (**Figure 2**). From a regional view, the number of patients varies according to prefectures in Japan. In the 2011 and 2012 epidemic period, the numbers of patients increased in most of the prefectures compared to those in previous years (**Figure 3**). Approximately 80% of *M. pneumoniae* pneumonia patients in Japan are in the 1–14-year-old age group, although *M. pneumoniae* pneumonia occurs in all age groups.

The national surveillance of *M. pneumoniae* pneumonia in the NESID program is based on reports from sentinel hospitals, and is thus not a survey of the total number of patients with this disease. Since the sentinels are large hospitals, information of *M. pneumoniae* pneumonia patients who are diagnosed and treated in small outpatient clinics is not included in the NESID data. The NESID also does not provide molecular epidemiological data or drug resistance information of *M. pneumoniae* clinical isolates. Although these issues need to be addressed, the weekly surveillance data provided by the NESID is nevertheless useful and functions as an alert for public health workers, medical institutions, and researchers. The NESID data allow for researchers to conduct detailed epidemiological studies to grasp the actual situation of *M. pneumoniae* infections, including molecular epidemiological and drug resistance aspects of this infectious disease, especially when the signs of epidemics are observed in the surveillance data.

## Molecular Epidemiology

**FIGURE 4:**
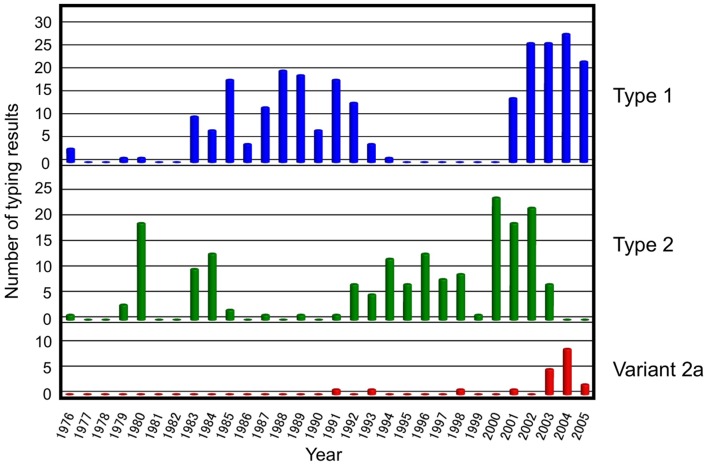
**Typing analysis of the *M. pneumoniae p1* gene from isolates detected in Japan between 1976 and 2005.** The majority of *M. pneumoniae* strains and clinical specimens included in this analysis were collected in the Kanagawa prefecture. Figure adapted from Kenri et al. (2008).

In general, *M. pneumoniae* clinical isolates can be classified into two distinct genetic lineages (type 1 and 2 lineages) based on their genomic background (Simmons et al., 2013; Brown et al., 2015; Lluch-Senar et al., 2015; Touati et al., 2015; Xiao et al., 2015). The *p1* gene, which encodes the major cytadhesin P1 protein, exhibits sequence polymorphism between the type 1 and 2 lineages (Su et al., 1990; Kenri et al., 1999; Spuesens et al., 2010; Zhao et al., 2011; Dumke et al., 2015). The *p1* types were also referred as the *p1* subtypes or groups in previous studies. A number of typing analyses of *M. pneumoniae* isolates based on *p1* gene polymorphism have been reported to date (Jacobs et al., 1996; Sasaki et al., 1996; Cousin-Allery et al., 2000; Kenri et al., 2008; Dumke et al., 2010b; Liu et al., 2010; Martinez et al., 2010). In our previous study, we genotyped *M. pneumoniae* isolated mainly in Kanagawa prefecture, Japan, and found that the rate of *p1* types 1 and 2 detection was not constant but rather varied year by year (**Figure 4**) (Sasaki et al., 1996; Kenri et al., 2008). In brief, type 2 was dominant in the early 1980s, 1990s, and at the beginning of the 2000s, while type 1 was dominant in the late 1980s and after 2003. This study demonstrated an alternative type-shift phenomenon of *M. pneumoniae p1* types with intervals of about 10 years. The time dependency of the rate of types 1 and 2 of the *p1* gene on the study period has also been observed in isolates from other parts of the world; however, no reports have shown a clear type-shift pattern similar to that observed in Kanagawa prefecture (Cousin-Allery et al., 2000; Pereyre et al., 2007; Martinez et al., 2010; Spuesens et al., 2012; Diaz et al., 2015; Kogoj et al., 2015). In typing analyses of isolates in Germany, the rate of types 1 and 2 detection was found to be relatively constant during the research period of about 10 years including the epidemics in 2011–2013 (Dumke et al., 2010b, 2015; Jacobs et al., 2015). The relationship between *M. pneumoniae* pneumonia epidemics and two type lineages of *M. pneumoniae* remains unclear (Jacobs et al., 1996, 2015; Kenri et al., 2008).

According to several *p1* typing studies, the dominant *M. pneumoniae* in Japan has been considered to be type 1 strains since 2003 (Fujii et al., 2012; Horino, 2012; Ohya et al., 2012; Ishiguro et al., 2015; Kubota et al., 2015; Suzuki et al., 2015). Apparent *p1* type shift of *M. pneumoniae* clinical strains was not observed during the large epidemic period in 2011 and 2012. For example, 126 *M. pneumoniae* strains were isolated at the Kanagawa Prefectural Institute of Public Health between 2003 and 2011. Of these, 101 (80%) were type 1, 7 (6%) were type 2, and 18 (14%) were type 2 variants (Ohya et al., 2012). At the Yamagata Prefectural Institute of Public Health, 358 isolates were genotyped between 2004 and 2013. Of these, 278 (77.7%) were type 1, 10 (2.8%) were variant 2a, 5 (1.4%) were variant 2b, and 65 (18.2%) were variant 2c. No type 2 strain was detected in Yamagata. In the case of Yamagata, type 1 accounted for 85–100% of the annual isolates recorded between 2004 and 2011. However, the isolation rate of type 1 reduced to 73.5% (83/113) and 33.9% (21/62) in 2012 and 2013, respectively. The proportion of variant 2c isolates increased in 2012 and 2013, as a counterpart of type 1 (Suzuki et al., 2015). At this point, it is not clear whether this is the sign of *p1* type change in this area. Although type 1 is considered to be dominant across a wide area of Japan at present, there is a report of a local area where type 2 is prevalent (Ishiguro et al., 2015). Most of type 2 lineage strains isolated in Japan in recent years are variants 2a and 2c while variant 2b and type 2 strains are rare. Type 2 was frequently found among the clinical isolates before 2000 (**Figure 4**), but type 2 was replaced by its variants (2a and 2c) almost completely during the last decade. The dominance of type 1 was also reported in recent clinical isolates in China and France (Liu et al., 2010; Wang et al., 2012; Pereyre et al., 2013; Tian et al., 2013; Zhao et al., 2013a; Xiao et al., 2014; Xue et al., 2014; Zhou et al., 2015). The results of a recent study conducted in Beijing, China indicate an increasing trend of type 2 lineage (Zhao et al., 2015), although it is not yet clear whether the type-shift phenomenon from type 1 to 2 is occurring. Further *p1* typing analysis is needed to explore whether type 2 lineage strains might become prevalent in the future in areas where type 1 are currently dominant.

Multilocus variable-number tandem-repeat analysis (MLVA)-based typing is a newly developed strategy for molecular epidemiological analyses of *M. pneumoniae* (Degrange et al., 2009; Chalker et al., 2015). Several reports demonstrated that Japanese clinical isolates can be also separated into multiple MLVA types as same as the clinical strains isolated in the other areas (Degrange et al., 2009; Kubota et al., 2015; Touati et al., 2015). However, more MLVA typing studies are needed to discuss the characteristic profiles of Japanese strains in terms of this typing method.

## Serological Characterization of *M. pneumoniae* Pneumonia Patient Sera Against Type 1 and 2 P1 Proteins

**FIGURE 5:**
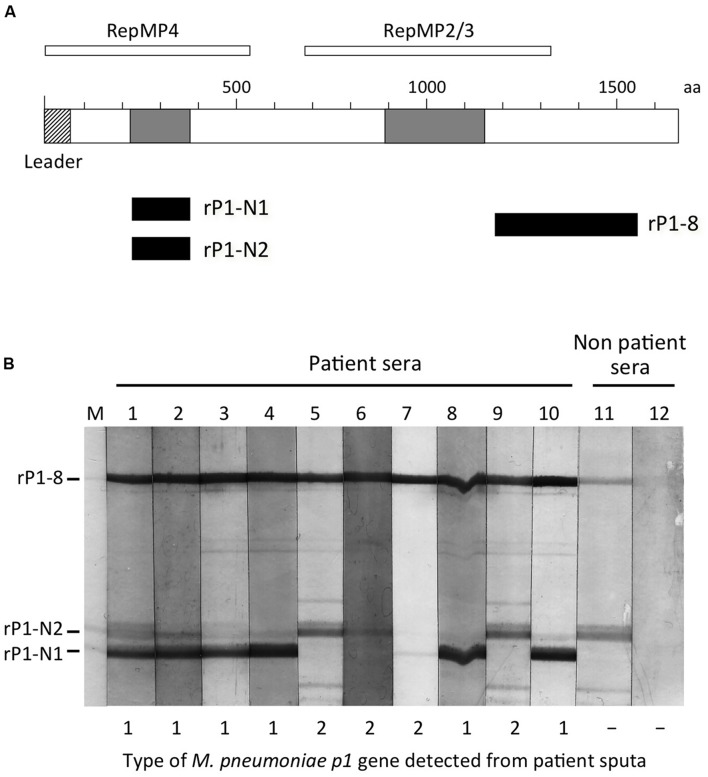
**Western blotting of the sera from *M. pneumoniae* pneumonia patients against recombinant P1 antigens. (A)** Schematic illustration of the P1 protein structure. P1 is a 170-kDa membrane protein consisting of about 1,630 amino acids (aa) depending on the strains. The first 59 aa of this protein (hatched box) is a leader peptide, which is removed during maturation. The two gray boxes indicate polymorphic regions of P1 protein that exhibit amino acid sequence variation between type 1 and 2 strains. RepMP4 and RepMP2/3 indicate repetitive regions of the *p1* gene. Multiple copies of DNA sequences similar to the RepMP4 and RepMP2/3 regions are present throughout the *M. pneumoniae* genome. The three filled boxes indicate the positions of recombinant P1 protein fragments produced in *E. coli*. The rP1-N1 region corresponds to aa 218 to 352 of the P1 protein of M129 strain (type 1). The rP1-N2 region corresponds to aa 218 to 357 of the P1 protein of FH strain (type 2). The rP1-8 region corresponds to aa 1160 to 1518 of M129 P1 (Kenri et al., 2006b). **(B)** Western blotting of patient sera against rP1-N1, rP1-N2, and rP1-8 recombinant proteins. Purified rP1-N1, rP1-N2, and rP1-8 proteins were separated by 10% sodium dodecyl sulfate-polyacrylamide gel electrophoresis and were blotted on a nitrocellulose membrane. Diluted (100-fold) serum samples from nine patients and two healthy subjects were reacted with the membrane. IgGs bound to the membrane were detected by alkaline phosphatase-conjugated anti-human IgG secondary antibody. The positions of recombinant P1 protein fragments are shown on the left. Lane M: Coomassie Brilliant Blue stain of the blotted membrane; lanes 1–10: patient sera (serum samples of lanes 5 and 9 were obtained from the same patient in an interval of 1 week); lanes 11 and 12: serum from healthy subjects. Types of *p1* genes detected by the nested PCR method (Kenri et al., 2008) from the sputum of same patient are shown on the bottom. The analyses of serum and sputum samples were performed as a part of previous studies (Yamazaki et al., 2006; Kenri et al., 2008).

P1 cytadhesin is one of the major antigens of *M. pneumoniae* that induce antibody production. Indeed, the anti-P1 antibody is frequently detected in the sera of *M. pneumoniae* pneumonia patients (Hirschberg et al., 1991; Razin and Jacobs, 1992; Rastawicki et al., 1996; Tuuminen et al., 2001). Since the P1 protein exhibits amino acid sequence polymorphism between the type 1 and 2 lineages, there was a possibility that P1 proteins of types 1 and 2 have different immunogenicity and induce specific antibodies during infection. In support of this idea, there are reports of the production of a monoclonal antibody that specifically recognizes type 1 P1 protein (Gerstenecker and Jacobs, 1990; Jacobs et al., 1996), and induction of type-specific immunity was achieved with P1 fragment antigens in guinea pigs (Dumke et al., 2008). To obtain more evidence for specific immunological responses to the two P1 types, we engineered and produced three recombinant P1 protein fragments, shown in **Figure 5A**. The amino acid sequences of the rP1-N1 and rP1-N2 regions are derived from P1 protein of the strains M129 (type 1) and FH (type 2), respectively (Kenri et al., 2006a,b). These regions exhibit the highest differences between type 1 and 2 P1 proteins, whereas the amino acid sequence of rP1-8 region is identical between type 1 and 2 P1 proteins. Using these three P1 fragments as antigens, we performed a western blotting analysis of the sera of nine *M. pneumoniae* pneumonia patients to detect anti-P1 IgG (**Figure 5B**). The patient sera chosen for this western blot were those that exhibited high antibody titers in the serological diagnosis for *M. pneumoniae* pneumonia (see **Table 2**). Furthermore, *p1* genes were detected from sputum samples of the same patients and were genotyped by PCR (**Figure 5B** and **Table 2**). This information indicates the most probable type of *M. pneumoniae* that infected the patients. The western blot result is shown in **Figure 5B**. All of the patient sera exhibited similar reactivity against the rP1-8 fragment, whereas the reactivity against rP1-N1 and rP1-N2 varied depending on the serum sample (**Figure 5B**). The sera from patients whose sputa were type 1 *p1*-positive in PCR showed stronger reactivity against rP1-N1 (**Figure 5B**, lanes 1–4, 8, and 10). On the other hand, the sera from patients with type 2 *p1*-positive sputa exhibited stronger reactivity against rP1-N2 (**Figure 5B**, lanes 5, 6, and 9) except for one serum (**Figure 5B**, lane 7). One of the serum samples from a healthy subject, used as a negative control, also showed weak reactivity against rP1-8 and rP1-N2 (**Figure 5B**, lane 11). This individual most likely had a previous, and perhaps unnoticed, infection with type 2 *M. pneumoniae*. These results demonstrated that induction of type-specific anti-P1 antibodies occurs in humans during *M. pneumoniae* infection (Kenri et al., 2006a,b).

**Table 2:** Hemadsorption inhibitory activity of patient and non-patient sera.

Serum No.^a^	Antibody titer^b^	*p1* gene types detected from patient sputa^c^	Hemadsorption (HA) inhibitory activity^d^			
			M129 (Type 1)		FH (Type 2)	
			1/5	1/10	1/5	1/10
1	1280	1	+	±	–	–
2	2560	1	+	±	±	–
3	1280	1	+	+	+	±
4	>2560	1	+	+	–	–
5	>2560	2	±	–	+	±
6	>2560	2	±	–	+	±
7	1280	2	±	–	±	–
8	>2560	1	+	+	–	–
9	1280	2	±	–	+	±
10	>2560	1	+	–	–	–
11	ND	NT	–	–	–	–
12	ND	NT	–	–	–	–

*^a^Serum number corresponds to the lane number of **Figure 5B**. ^b^Antibody titer was measured with Serodia Myco II kit (Fujirebio, Tokyo, Japan); ND, not determined. ^c^Detection and typing of the p1 gene was performed by the nested PCR method as a part of previous study (Kenri et al., 2008); NT, not tested. ^d^Five to ten colonies of M. pneumoniae M129 (type 1) or FH (type 2) strains were formed on PPLO agar cast in a 24-well microplate (1.5 ml PPLO agar per well). Colony-forming wells were soaked with 1 ml of phosphate-buffered saline (PBS) for 5 min at room temperature. After removal of PBS, 0.1 ml of diluted patient serum (fivefold or 10-fold dilution by PBS) was added to the well and incubated for 20 min at 37°C. After removal of the patient serum, 0.5 ml of a diluted sheep red blood cell (RBC) suspension (100-fold dilution by PBS) was added to the well and incubated for 20 min at 37°C. After incubation, excess RBCs were removed by washing with 1 ml of PBS three times. The state of hemadsorption of M. pneumoniae colonies was evaluated by microscopic observation. +: complete hemadsorption inhibition (no adsorption of RBCs was observed); ±: partial inhibition (partial adsorption of RBCs was observed); –: no inhibition (colonies were fully covered by RBCs; Kenri et al., 2006b).*

## Hemadsorption (HA) Inhibitory Activity of *M. pneumoniae* Pneumonia Patient Sera

It has been reported that *M. pneumoniae* pneumonia patient sera exhibit inhibitory activity toward the adsorption of red blood cells to *M. pneumoniae* colonies [i.e., hemadsorption (HA) inhibitory activity; Hirschberg et al., 1991; Razin and Jacobs, 1992; Rastawicki et al., 1996; Tuuminen et al., 2001; Schurwanz et al., 2009]. Thus, we examined the HA inhibitory activity of the patient sera analyzed by western blotting in **Figure 5B**, and the results are shown in **Table 2**. The sera from type 1-infected patients (No. 1–4, 8, and 10) tended to show stronger HA inhibitory activity against M129 strain (type 1). On the other hand, the sera from type 2-infected patients (No. 5, 6, and 9) exhibited stronger HA inhibitory activity against FH strain (type 2) compared to M129. This result indicated that *M. pneumoniae* pneumonia patient sera with a high antibody titer possessed type-specific HA inhibitory activities (Kenri et al., 2006a,b). Indeed, some anti-P1 antibodies have been reported to show HA inhibitory activity (Krause and Baseman, 1983; Jacobs et al., 1989; Gerstenecker and Jacobs, 1990); however, it is not clear whether the type-specific anti-P1 antibodies detected in the western blotting in **Figure 5B** play a role in determining the type-specific HA inhibitory activity. It is possible that antibodies against another type-specific antigen are responsible for the observed type-specific HA inhibitory activity. If these type-specific HA inhibitory activities of patient sera serve as protective immunity for *M. pneumoniae* infection, this might explain the type shift phenomenon of *M. pneumoniae* isolates.

## Emergence of Macrolide-Resistant *M. pneumoniae* (MRMP)

It has been known at least since the 1970s that *M. pneumoniae* can acquire resistance against macrolides relatively easily in laboratory culture (Niitu et al., 1970; Nitu et al., 1974; Lucier et al., 1995; Pereyre et al., 2004). There are also some early reports of MRMP clinical isolates (Stopler et al., 1980; Clara et al., 1989). However, MRMPs rapidly and broadly spread after 2000, especially in eastern Asian countries such as China, Korea, and Japan (Okazaki et al., 2001; Matsuoka et al., 2004; Morozumi et al., 2005, 2008; Cao et al., 2010; Bebear et al., 2011; Hong et al., 2013; Zhao et al., 2013b, 2014). The frequencies of MRMP detection are now increasing in other areas of the world (Pereyre et al., 2007; Li et al., 2009; Peuchant et al., 2009; Dumke et al., 2010a; Averbuch et al., 2011; Chironna et al., 2011; Yamada et al., 2012; Eshaghi et al., 2013; Tsai et al., 2013; Wu et al., 2013; Caballero Jde et al., 2014; Saraya et al., 2014; Diaz et al., 2015; Zheng et al., 2015). MRMPs are isolated more frequently from adolescent and pediatric patients than from adults, which is likely related to the frequent use of macrolides for treatments of mycoplasmal infections at younger ages. However, isolation of MRMPs from adult patients is also on the rise (Isozumi et al., 2009; Miyashita et al., 2012; Yoo et al., 2012; Hanada et al., 2014; Zhou et al., 2015). Most of the MRMPs isolated in Japan carry the A2063G mutation in domain V of the 23S rRNA gene that confers strong resistance to 14- and 15-membered macrolides and lincosamides. However, there is a report of a local outbreak caused by MRMPs carrying the A2063T mutation, which exhibits only moderate resistance to macrolides (Suzuki et al., 2013). The most recent estimate of the isolation rate of MRMPs from adolescent and pediatric patients in Japan is 50–90%, depending on the area (Morozumi et al., 2008; Akaike et al., 2012; Miyashita et al., 2012; Kawai et al., 2013b; Ishiguro et al., 2015). Given this situation, the Japan Pediatric Society^3^ and The Japanese Society for Mycoplasmology^4^ have issued guiding principles for treating *M. pneumoniae* pneumonia (The committee on the guiding principle for treatment of *Mycoplasma pneumoniae* pneumonia, 2014).

## Therapeutic Strategies for *M. pneumoniae* Pneumonia

*Mycoplasma pneumoniae* is generally susceptible to macrolides, tetracyclines, and the new quinolone antibiotics. However, as stated above, the emergence of MRMP since 2000 has made the treatment of *M. pneumoniae* pneumonia challenging. Although MRMP strains have been reported in European countries and in the United States, the detection rates from these countries are lower than those in East Asia, including Japan. Caution in monitoring and treating MRMP strains and the necessity of continuous surveillance for these strains are partially described in the guidelines of the Infectious Diseases Society of America (IDSA), American Thoracic Society (ATS; Mandell et al., 2007), European Respiratory Society, European Society for Clinical Microbiology and Infectious Diseases for adults (Woodhead et al., 2011), and in the Pediatric Infectious Diseases Society (PIDS) and IDSA for children (Bradley et al., 2011). However, the necessity and strategies of alternative antibiotic treatment for MRMP strains have not been described in detail. The detection rates of MRNPs are associated with age (Miyashita et al., 2013): detection rates are higher in children aged ≤15 years than in adults. In addition, the detection rates among adults are higher in adolescents aged 16–19 years than in those aged ≥20 years.

The guidelines of the IDSA and ATS recommend macrolides or tetracyclines as the first-line drugs for *M. pneumoniae* pneumonia, and fluoroquinolones as the second-line drugs in adults. The guidelines of the PIDS and IDSA for children recommend azithromycin as the first-line oral drug, and clarithromycin, erythromycin, or doxycycline (for patients aged ≥8 years) along with levofloxacin or moxifloxacin (for adolescent patients) as the second-line oral drugs, for mild cases. For treatment via injection, azithromycin is recommended as the first-line drug (although this is not indicated for children in Japan), and erythromycin and levofloxacin (also not indicated for children in Japan) are recommended as the second-line drugs.

Thus, the applicability of antibiotics differs between adults and children; in addition, the indication for antibiotics for children is different between Japan and other countries. In view of these points, the therapeutic guiding principles^3,4^ for *M. pneumoniae* pneumonia issued in Japan are reviewed in the following sections.

### Treatment in Adults: Recommendations by the Japanese Society of Mycoplasmology

The first-line drugs for *M. pneumoniae* pneumonia in adults are macrolide antibiotics. Oral administration of clarithromycin (400 mg/day administered in two divided doses) or erythromycin (800–1,200 mg/day administered in 4–6 divided doses for 7–10 days) is recommended for patients on an outpatient basis. Oral azithromycin administered at 500 mg once a day for 3 days or at 2 g once a day for 1 day is also indicated (**Table 3**). The minimum inhibitory concentration (MIC) values of macrolides for *M. pneumoniae* without macrolide resistance genes are extremely low, while those of fluoroquinolones and tetracyclines are higher (Akaike et al., 2012). Minocycline, levofloxacin, garenoxacin, moxifloxacin, sitafloxacin, and tosufloxacin are recommended as second-line drugs (**Table 3**). Intravenous administration is indicated for inpatients: minocycline, azithromycin, and erythromycin are recommended as first-line drugs, and levofloxacin, ciprofloxacin, and pazufloxacin are recommended as the second-line drugs (**Table 4**).

**Table 3:** Recommended treatments for adult outpatients of *M. pneumoniae* pneumonia.

	Drug	Route of administration	mg/dose	Dose/day
First-line drug	Clarithromycin (CAM)	Oral	200	2
	Azithromycin (AZM) (Slow-release formulation)	Oral	2000	1 (1 day)
	Azithromycin (AZM)	Oral	500	1 (3 days)
	Erythromycin (EM)	Oral	200	4–6
Second-line drug	Minocycline (MINO)	Oral	100	2
	Levofloxacin (LVFX)	Oral	500	1
	Garenoxacin (GRNX)	Oral	400	1
	Moxifloxacin (MFLX)	Oral	400	1
	Sitafloxacin (STFX)	Oral	100	2
		Oral	200	1
	Tosufloxacin (TFLX)	Oral	150	2–3

*The table is adapted from the guiding principle (Chiryo–Shishin) issued by the Japanese Society of Mycoplasmology (JSM). http://square.umin.ac.jp/jsm/shisin.pdf*.

**Table 4:** Recommended treatments for adult inpatients of *M. pneumoniae* pneumonia.

	Drug	Route of administration	mg/dose	Dose/day
First-line drug	Minocycline (MINO)	Intravenous (drip infusion)	100	2
	Azithromycin (AZM)	Intravenous (drip infusion)	500	1
	Erythromycin (EM)	Intravenous (drip infusion)	300–500	2–3
Second-line drug	Levofloxacin (LVFX)	Intravenous (drip infusion)	500	1
	Ciprofloxacin (CPFX)	Intravenous (drip infusion)	300	2
	Pazufloxacin (PZFX)	Intravenous (drip infusion)	500–1000	2

*The table is adapted from the guiding principle of JSM (http://square.umin.ac.jp/jsm/shisin.pdf*).

There is concern surrounding the emergence of quinolone resistance in *Streptococcus pneumoniae* and other respiratory pathogenic bacteria owing to the use of quinolones as the initial treatment for pneumonia. Quinolones have been shown capable of inducing resistance in *M. pneumoniae* strains *in vitro* (Gruson et al., 2005). Therefore, quinolones should be avoided during the initial treatment in young patients with suspected *M. pneumoniae* pneumonia. *M. pneumoniae* pneumonia is less common in the elderly than in the younger generation. Quinolones are prescribed for elderly patients with pneumonia, because microorganisms other than *M. pneumoniae* are typically involved. On the other hand, tetracyclines are not likely to induce resistance in *M. pneumoniae*. Intravenous administration of tetracycline is recommended as the first-line treatment for hospitalized patients (**Table 4**). Although there is no sufficient evidence regarding the duration of antibiotic administration for the treatment of *M. pneumoniae* pneumonia in adults, the Japanese Society of Mycoplasmology recommends a duration of 7–10 days.

When the fever does not subside within 48–72 h after the administration of macrolides, the antibiotics should be changed considering the possibility of MRMP involvement. At present, MRMP is susceptible to both tetracyclines and fluoroquinolones. A study of the treatment for infection with MRMP in children demonstrated that minocycline showed a higher rate of bacterial elimination than tosufloxacin, and the fever subsided faster upon treatment with minocycline than with tosufloxacin (Kawai et al., 2013a).

Considering the emergence of quinolone resistance, the first-line drug recommended for MRMP pneumonia is minocycline (**Table 5**). Oral minocycline administered at 200 mg/day in two divided doses on an outpatient basis, and intravenous minocycline administered at 100 mg twice a day for inpatients is recommended. Quinolones are recommended as second-line drugs for both outpatients and inpatients (**Tables 5** and **6**). For outpatient treatment, levofloxacin, garenoxacin, moxifloxacin, sitafloxacin, or tosufloxacin is orally administered (**Tables 3** and **5**). For inpatient treatment, levofloxacin, ciprofloxacin, or pazufloxacin is intravenously administered (**Tables 4** and **6**). The duration of antibiotic treatment is 7–10 days, similar to that for infection with macrolide-sensitive *M. pneumoniae*.

**Table 5:** Recommended treatments for adult outpatients of macrolide-resistant *M. pneumoniae* pneumonia.

	Drug	Route of administration	mg/dose	Dose/day
First-line drug	Minocycline (MINO)	Oral	100	2
Second-line drug	Levofloxacin (LVFX)	Oral	500	1
	Garenoxacin (GRNX)	Oral	400	1
	Moxifloxacin (MFLX)	Oral	400	1
	Sitafloxacin (STFX)	Oral	100	2
			200	1
	Tosufloxacin (TFLX)	Oral	150	2–3

*The table is adapted from the guiding principle of JSM (http://square.umin.ac.jp/jsm/shisin.pdf*).

**Table 6:** Recommended treatments for adult inpatients of macrolide-resistant *M. pneumoniae* pneumonia.

	Drug	Route of administration	mg/dose	Dose/day
First-line drug	Minocycline (MINO)	Intravenous (drip infusion)	100	2
Second-line drug	Levofloxacin (LVFX)	Intravenous (drip infusion)	500	1
	Ciprofloxacin (CPFX)	Intravenous (drip infusion)	300	2
	Pazufloxacin (PZFX)	Intravenous (drip infusion)	500–1000	2

*The table is adapted from the guiding principle of JSM (http://square.umin.ac.jp/jsm/shisin.pdf*).

In severe cases of *M. pneumoniae* pneumonia involving respiratory failure, methylprednisolone is administered at 500–1,000 mg/day for 3–5 days concomitantly with an appropriate antimycoplasmal drug. It should be noted that the relationship between MRMP and severe *M. pneumoniae* infection remains unclear.

### Treatment in Children: Recommendations by the Japanese Society of Pediatric Pulmonology, Japanese Society for Pediatric Infectious Diseases, Japan Pediatric Society, and Japanese Society of Mycoplasmology

Macrolides are also recommended as the first-line drugs for *M. pneumoniae* pneumonia in children. The incidence of MRMP isolates from pediatric cases varies both regionally and epidemically in Japan, ranging from <50% in patients without a history of macrolide treatment to >90% in patients that were treated by macrolides without clinical effectiveness. The MIC values of macrolides for macrolide-sensitive *M. pneumoniae* are extremely low, and the rates of bacterial elimination are high. The MIC values of tetracyclines and tosufloxacin for macrolide-sensitive *M. pneumoniae* are relatively higher than those of macrolides. In addition, *M. pneumoniae* may not be eliminated after treatment with these drugs. Tosufloxacin is the only fluoroquinolone approved for children in Japan (**Table 7**).

**Table 7:** Recommended treatments for pediatric patients of *M. pneumoniae* pneumonia.

Drug	Route of administration	Drug dose (mg/kg/day)	Divided dose/day	Treatment period (days)
Erythromycin (EM)	Oral	25–50	4–6	14
Clarithromycin (CAM)	Oral	10–15	2–3	10
Azithromycin (AZM)	Oral	10	1	3
Tosufloxacin (TFLX)	Oral	12	2	7–14
Minocycline (MINO)	Oral or intravenous drip infusion	2–4	2	7–14

*The table is adapted from the guiding principle of JSM (http://square.umin.ac.jp/jsm/shisin.pdf*).

In children with pneumonia caused by macrolide-sensitive *M. pneumoniae*, treatment with macrolides has been shown to alleviate the fever within 48 h in more than 80% of cases. On the other hand, fever persists in approximately 70% of children with MRMP infection. These data indicate that the clinical effectiveness of macrolides should be evaluated on the basis of the presence of fever at 48–72 h after treatment. Persistent fever suggests the possibility of infection with macrolide-resistant strains or mixed infection with microorganisms other than *M. pneumoniae*. For cases of *M. pneumoniae* pneumonia with failure of macrolide treatment, either tosufloxacin or tetracyclines are applicable. Quinolones might induce drug resistance in the microflora other than *M. pneumoniae*, and should be administered with caution. Tetracyclines may cause side effects such as transient anostosis, staining of the teeth, and enamel hypoplasia. Tetracyclines are contraindicated in children aged under 8 years. The recommended duration of treatment for *M. pneumoniae* pneumonia caused by macrolide-sensitive strains is 14 days with erythromycin, 10 days with clarithromycin, and 3 days with azithromycin. Treatment with tosufloxacin or tetracyclines for pneumonia caused by MRMPs should be administered for 7–14 days. Systemic administration of steroids for severe pneumonia should be considered; however, the indications of systemic steroid use are not yet determined. One study showed that steroid therapy is effective in patients with fever lasting >7 days, with a serum lactate dehydrogenase level of ≥480 IU/L (Oishi et al., 2011).

## Conclusion

The nationwide surveillance of *M. pneumoniae* pneumonia in Japan is based on reports collected from approximately 500 sentinel hospitals, and is thus not reflective of the total number of patients. However, this weekly monitoring can detect previous epidemics and patterns, and has played a significant role as an alert system for medical and public health workers as well as researchers. Therefore, the effort for this surveillance program should be continued.

There are two distinct genetic lineages of *M. pneumoniae* that exhibit polymorphism in the cytadhesin P1 protein sequence. However, the involvements of these two lineages in pneumonia epidemics and differences in their pathogenicity are not yet fully understood. Nevertheless, cytadhesin P1 is an important factor that plays a critical role in the infection mechanism of this pathogen and in the interactions with host cells. We believe that *p1* gene typing is an important aspect for molecular epidemiological studies of *M. pneumoniae* and should be performed by combining modern genotyping methods based on MLVA, multi-locus sequence typing, and/or whole-genome SNP strategies.

Emergence of MRMP is serious problem for the treatment of *M. pneumoniae* pneumonia. Given this situation, several Japanese academic and medical societies have issued specific guiding principles for treatment of *M. pneumoniae* pneumonia. Macrolides are still recommended as the first-line drug in children and adults. However, if the fever does not subside in 48–72 h from first-line drug administration, a change of antibiotics to second-line drugs (i.e., fluoroquinolones and tetracyclines) is recommended.

## Author Contributions

TY wrote the therapeutic strategy section of the paper. TK wrote the surveillance and epidemiology sections of the paper.

## Conflict of Interest Statement

The authors declare that the research was conducted in the absence of any commercial or financial relationships that could be construed as a potential conflict of interest.

## Footnotes

1 http://www.mhlw.go.jp/bunya/kenkou/kekkaku-kansenshou11/01-05-38.html

2 http://www.nih.go.jp/niid/ja/10/2096-weeklygraph/1659-18myco.html

3 http://www.jpeds.or.jp/uploads/files/saisin_130219_2.pdf

4 http://square.umin.ac.jp/jsm/shisin.pdf
